# Animal-adapted members of the *Mycobacterium tuberculosis* complex endemic to the southern African subregion

**DOI:** 10.4102/jsava.v87i1.1322

**Published:** 2016-04-26

**Authors:** Charlene Clarke, Paul van Helden, Michele Miller, Sven Parsons

**Affiliations:** 1 SAMRC Centre for TB Research, DST/NRF Centre of Excellence for Biomedical Tuberculosis Research, Division of Molecular Biology and Human Genetics, Faculty of Medicine and Health Sciences, Stellenbosch University, South Africa

## Abstract

Members of the *Mycobacterium tuberculosis* complex (MTC) cause tuberculosis (TB) in both animals and humans. In this article, three animal-adapted MTC strains that are endemic to the southern African subregion – that is, *Mycobacterium suricattae, Mycobacterium mungi*, and the dassie bacillus – are reviewed with a focus on clinical and pathological presentations, geographic distribution, genotyping methods, diagnostic tools and evolution. Moreover, factors influencing the transmission and establishment of TB pathogens in novel host populations, including ecological, immunological and genetic factors of both the host and pathogen, are discussed. The risks associated with these infections are currently unknown and further studies will be required for greater understanding of this disease in the context of the southern African ecosystem.

## Introduction

Tuberculosis (TB) is an infectious disease of humans and animals caused by bacteria belonging to the *Mycobacterium tuberculosis* complex (MTC). In addition to causing severe morbidity and mortality in humans, the disease impacts the agricultural industry as identification of infection in an animal population results in state-enforced implementation of quarantine and test-and-cull or depopulation control strategies (Huard *et al*. 2006).

**FIGURE 1:**
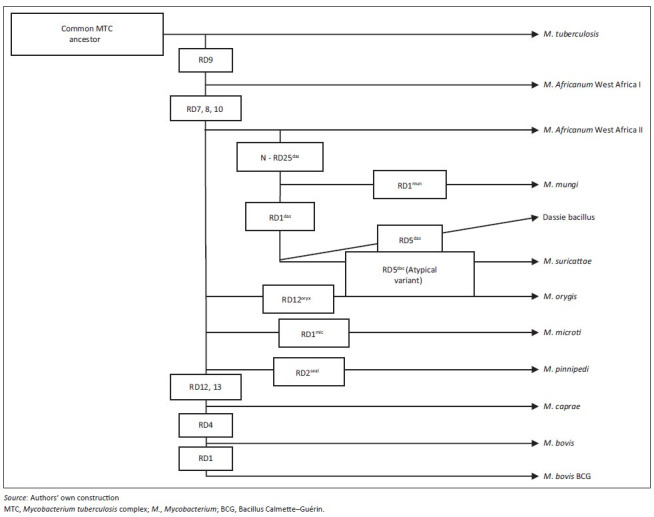
Phylogeny of the *Mycobacterium tuberculosis* complex indicating genetic regions of difference which differentiate between selected members.

The MTC consists of 11 members, many of which display apparent host adaptation, and one culture-adapted strain, that is, *Mycobacterium bovis* Bacillus Calmette–Guérin (Figure 1). These members share greater than 99% genetic similarity at the nucleotide level (Brosch *et al*. 2002; Mostowy, Cousins & Behr 2004) but differ notably in their host niche, geographic distribution and pathogenicity (Brosch *et al*. 2002; Huard *et al*. 2006). MTC strains associated with human infection are *Mycobacterium africanum, Mycobacterium canetti* and *M. tuberculosis*, the latter being the most significant cause of human TB (Comas *et al*. 2013; Huard *et al*. 2006). MTC members that cause TB in animal hosts include *M. bovis*, which affects numerous domestic and wildlife species, *Mycobacterium pinnipedii*, which typically infects pinniped species in the southern hemisphere, *Mycobacterium microti*, which is associated with small rodents, *Mycobacterium caprae*, which mainly causes TB in goats, and *Mycobacterium orygis*, which is associated with various species (Huard *et al*. 2006; Smith *et al*. 2006). Furthermore, three closely related members of the MTC have been isolated from southern African mammals, including the dassie bacillus, which infects rock hyraxes (*Procavia capensis*) (Mostowy *et al*. 2004), *Mycobacterium mungi*, which infects banded mongooses (*Mungos mungo*) (Alexander *et al*. 2010), and *Mycobacterium suricattae*, which infects meerkats (*Suricata suricattae*) (Parsons *et al*. 2013).

Whilst many of the MTC members display characteristic phenotypes, they are most accurately distinguished from one another using various genetic markers. These include single nucleotide polymorphisms (SNPs) in the *16S rDNA* and *gyrB* genes and elsewhere in the genome as well as large sequence polymorphisms referred to as regions of difference (RD) (Huard *et al*. 2006). MTC-specific genotyping methods include IS*6110*-restriction fragment length polymorphism (RFLP) typing, which identifies differences in copy numbers and genetic insertion sites of the IS*6110* sequence, spacer oligonucleotide typing (spoligotyping), which identifies the presence or absence of specific sequences located in the direct repeat region of the genome, and Mycobacterial Interspersed Repetitive Units – Variable Number of Tandem Repeats (MIRU-VNTR) typing, which detects variation in the number of genetic repeats at various loci throughout the genome (McLernon *et al*. 2010).

Here we review the three animal-adapted MTC members that are endemic to the southern African subregion, that is, *M. suricattae, M. mungi* and the dassie bacillus. This review focuses on the geographic distribution, culture methods, molecular and immunological diagnostic tools and evolution of these mycobacterial organisms as well as the clinical and pathological presentation of disease in their host species.

## Dassie bacillus

**FIGURE 2:**
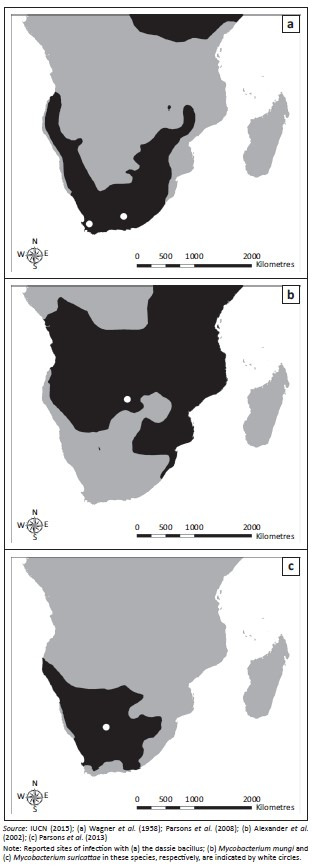
Geographic distribution in the southern hemisphere of Africa of (a) rock hyraxes (*Provacia capensis*); (b) banded mongooses (*Mungos mungi*) and (c) meerkats (*Suricata suricatta*).

The dassie bacillus was first isolated in 1954 from rock hyraxes in Nieu-Bethesda, Eastern Cape Province, South Africa (31°52.002’S, 24°33’E) (Wagner *et al*. 1958). More recently, cases of free-living rock hyraxes infected with this pathogen have been reported from the Groot Winterhoek Mountains, Western Cape Province (33°4.41’S, 19°9.78’E), suggesting that the organism may be widespread in the South African hyrax population (Figure 2a) (Parsons *et al*. 2008; Wagner & Bokkenheuser 1961). The bacillus has also been isolated from rock hyraxes and a meerkat that were captured from unknown locations in South Africa and first housed in London before being exported to Australia in 1989 (Cousins *et al*. 1994) and Sweden in 1990 (G. Bolske [Statens Veterinärmedicinska Anstalt, Sweden] pers. comm., 04 January 2007; Mostowy *et al*. 2004), respectively.

Cases of TB in rock hyraxes captured in South Africa and the United Arab Emirates have also been ascribed to *M. microti* (Lutze-Wallace *et al*. 2006) and *M. africanum* (Gudan *et al*. 2008), respectively. However, the organism described as *M. microti* in the former report had the same spoligotype as the dassie bacillus isolated from hyraxes in Australia (Lutze-Wallace *et al*. 2006; Mostowy *et al*. 2004). Moreover, the genotyping methods used to identify *M. africanum* in the latter study would not have differentiated between this strain and the dassie bacillus. Therefore, these cases may be further evidence of the widespread distribution of this organism.

Transmission of the dassie bacillus in rock hyraxes appears to be primarily via the respiratory tract, as the majority of TB lesions present as multiple whitish granulomas with caseous necrotic centres in the lung parenchyma and pleura (Cousins *et al*. 1994; Parsons *et al*. 2008; Wagner & Bokkenheuser 1961). Infection of the lungs is often accompanied by secondary spread to other organs, including the liver, kidneys, spleen and uterus, in which multifocal granulomas may be observed (Cousins *et al*. 1994; Parsons *et al*. 2008). The liver, spleen and inguinal and para-aortic lymph nodes may also be enlarged (Wagner & Bokkenheuser 1961). Animals can become emaciated (Cousins *et al*. 1994), but may also exhibit no apparent clinical signs despite the presence of significant disease (Parsons *et al*. 2008).

The dassie bacillus is a slow-growing *Mycobacterium*; cultures typically take 4–6 weeks to become detectible (Cousins *et al*. 1994; Smith 1960). Dorset egg medium without glycerol (Smith 1960, 1965; Wagner *et al*. 1958), Dubos medium with 0.1% glutamic acid (Cousins *et al*. 1994; Smith 1960) and BACTEC MGIT tubes (Becton Dickinson, Franklin Lakes, NJ, USA) (Parsons *et al*. 2008) have been used to culture the organism.

**FIGURE 3:**
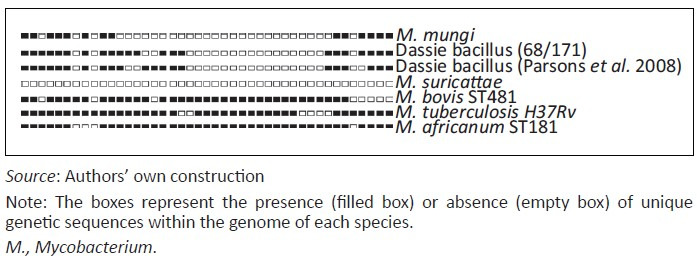
Spoligotype patterns of members of the *Mycobacterium tuberculosis* complex (MTC) occurring in the southern African subregion and representative patterns of other *Mycobacterium tuberculosis* complex species.

The dassie bacillus has a unique spoligotype (Figure 3) and MIRU-VNTR pattern (Table 1) and 10–15 copies of the IS*6110* sequence in its genome (Cousins *et al*. 1994). Furthermore, it has deletions of nine major RDs, including RD3, 7, 8, 9, 10, RD5^das^, RDVirS^das^, N-RD25^das^ and RD1^das^, as well as the SNP Rv1510^1129^ and the single nucleotide deletion Rv0911^389^ (Huard *et al*. 2006; Mostowy *et al*. 2004; Parsons *et al*. 2008). No immunological diagnostic tests for TB in hyraxes have been described.

**TABLE 1:** Representative Mycobacterial Interspersed Repetitive Units – Variable Number of Tandem Repeats patterns for the dassie bacillus, *Mycobacterium mungi, Mycobacterium suricattae and Mycobacterium africanum*.

Genomic locus	MIRU-VNTR repeat number†			

	*M. mungi*	*M. suricattae*	Dassie bacillus	*M. africanum*
MIRU 2	2	2	2	2
VNTR 424/Mtub04	3	3	2	4
VNTR 577/ETR-C	3	5	5	5
MIRU 4/ETR-D	3	2	3	2
MIRU 40	1	2	2	2
MIRU 10	5	6	7	7
MIRU 16	3	2	3	4
VNTR 1955/Mtub21	3	3	3	4
MIRU 20	2	2	2	2
VNTR 2163b/QUB11b	0	-	7	5
VNTR 2165/ETR-A	6	-	6	6
VNTR 2347/Mtub29	3	3	3	3
VNTR 2401/Mtub30	4	4	3	4
VNTR 2461/ETR-B	4	5	4	4
MIRU 23	4	4	4	4
MIRU 24	2	3	2	2
MIRU 26	4	4	5	4
MIRU 27	3	1	4	3
VNTR 3171/Mtub 34	3	3	3	3
MIRU 31/ETR-E	8/9	5	5	5
VNTR 3690/Mtub 39	-	8	5	4
VNTR 4052/QUB 26	-	3	4	6
VNTR 4156/QUB 4156	-	1	3	3
MIRU 39	2	2	2	2

*Source*: Reproduced from Parsons, S.D.C., Drewe, J.A., Gey van Pittius, N.C., Warren, R.M. & Van Helden, P.D., 2013, ‘Novel cause of tuberculosis in meerkats, South Africa’, *Emerging Infectious Diseases* 19, 2004–2007, with permission from Emerging Infectious Diseases

Note: These patterns allow for genetic differentiation between these strains.

*M., Mycobacterium*; MIRU-VNTR, Mycobacterial Interspersed Repetitive Units – Variable Number of Tandem Repeats.

† The number of repeats of genetic copies at the corresponding genomic locus.

## Mycobacterium mungi

TB in banded mongooses was first identified in the Chobe National Park, Botswana (17°49.33’S, 25°07.58’E), in 2000 (Alexander *et al*. 2010) (Figure 2b). On this occasion the causative agent was identified as *M. tuberculosis* (Alexander *et al*. 2002). However, a subsequent study described the aetiology of this disease as a novel MTC member which was named *M. mungi* (Alexander *et al*. 2010). Between 2000 and 2010, seven outbreaks of TB have been reported in banded mongooses in the park and the disease has caused high levels of mortality and threatened the survival of smaller groups (Alexander *et al*. 2010).

Unlike other members of the MTC, transmission of *M. mungi* is postulated to occur via environmental contamination. Bacteria enter the host through erosions of the nasal planum and once infection is established, it spreads systemically via lymphatic or haematogenous routes (Alexander *et al*. 2010). Disease presents as granulomas in the liver, spleen, lymph nodes and lungs, and enlargement of the liver and spleen may be observed (Alexander *et al*. 2002). Clinical signs in infected animals include anorexia, lethargy, generalised weakness, discoloration of the fur, nasal distortion, sneezing, enlarged mesenteric lymph nodes and enlarged testicles (Alexander *et al*. 2010, 2002; Fairbanks, Hawley & Alexander 2014). Behavioural changes may also be seen as infected animals show a lack of fear of humans (Alexander *et al*. 2002). Mongooses with advanced stages of TB may have increased faecal glucocorticoid metabolite levels (Laver *et al*. 2012) and death usually occurs within 2–3 months after the onset of clinical signs (Alexander *et al*. 2010).

*Mycobacterium mungi* has been cultured on pyruvate-enriched Lowenstein-Jensen slants with very few colonies visible after 5 to 6 weeks (Alexander *et al*. 2002). The spoligotype pattern of this organism is similar to that of the dassie bacillus (Figure 3) and MIRU-VNTR patterns can be used to distinguish this strain from other MTC members (Table 1) (Alexander *et al*. 2010). The *M. mungi* genome contains the N-RD25^das^ deletion and a unique RD1^das^ deletion that is larger than that of the dassie bacillus. No immunological diagnostic assays have been reported for detection of TB in mongooses.

## Mycobacterium suricattae

Tuberculosis was first observed in free-living meerkats from the Kalahari Desert in the 1990s (Kalahari Meerkat Project n.d.) (Figure 2c) and the causative agent was identified as *M. tuberculosis* (Alexander *et al*. 2002). Subsequent cases of TB in meerkats from the Kalahari Meerkat Project (KMP), South Africa (26°58’S, 21°49’E) have been attributed to *M. bovis* (Drewe *et al*. 2009a). However, more recently, the aetiology of TB in this population has been described as a novel MTC member named *M. suricattae* (Parsons *et al*. 2013). TB in meerkats from the KMP has resulted in the death of over 200 meerkats and the extinction of approximately 20 social groups (Meerkats Wiki n.d.).

The social behaviour of meerkats may influence the transmission of *M. suricattae* within a group with infection occurring primarily via the respiratory route (Drewe 2010; Drewe *et al*. 2009a). However, transmission may also occur via ingestion of bacteria from bite wounds or discharging skin wounds. Grooming and aggression have been regarded as important contributors to transmission between individuals (Drewe 2010; Drewe *et al*. 2011).

TB in meerkats is a disseminated disease affecting various organs with infection spreading via lymphatic or haematogenous routes (Drewe *et al*. 2009a). Granulomas are typically observed in the lungs, spleen, liver, mediastinal and submandibular lymph nodes (Drewe *et al*. 2009a). The most common clinical signs include enlargement of the submandibular lymph nodes, lethargy, emaciation, dyspnoea and enlarged axillary lymph nodes. The onset of clinical signs invariably leads to a fatal outcome for the infected animals (Alexander *et al*. 2002).

Mycobacterial isolates from meerkat TB lesions grown on Lowenstein-Jensen slants enriched with pyruvate resulted in few colonies after 5–6 weeks (Alexander *et al*. 2002) and required up to 10 weeks of culture for adequate growth (Drewe *et al*. 2009a). *Mycobacterium suricattae* has been cultured in BACTEC MGIT media, but only one of four isolates cultured on oleic albumin dextrose catalase-enriched Difco Middlebrook 7H10 agar resulted in sufficient growth for deoxyribonucleic acid (DNA) extraction (Parsons *et al*. 2013).

*Mycobacterium suricattae* is unique amongst the members of the MTC in that it has no spoligotype pattern (Figure 3), which is consistent with the deletion of the direct repeat region from this strain (Dippenaar *et al*. 2015; Parsons *et al*. 2013). The single isolate that has been comprehensively genetically characterised had 21 copies of the IS*6110* insertion sequence and its MIRU-VNTR patterns can be used to distinguish it from both *M. mungi* and the dassie bacillus (Table 1) (Parsons *et al*. 2013). The *16S rDNA* sequence of *M. suricattae* differs from all other MTC members with a T to G SNP at position 214 (*16S rDNA*^214^) (Dippenaar *et al*. 2015; Parsons *et al*. 2013). RD5^das^ has been deleted from the *M. suricattae* genome; however, this region differs from that in the dassie bacillus in containing an IS*6110* sequence in the forward orientation. *Mycobacterium suricattae* also has deletions of RD1^das^, N-RD25^das^ and RDVirS^das^ and the SNPs Rv1510^1129^ and Rv0911^389^ (Dippenaar *et al*. 2015; Parsons *et al*. 2013). Additional unique and characteristic RDs have recently been identified following the whole genome sequencing of *M. suricattae* and include RD900 which, as for *M. africanum,* has not been deleted from this strain (Dippenaar *et al*. 2015).

Serological diagnostic assays, such as the multi-antigen print immunoassay and BovidTB Stat-Pak Assay (Chembio Diagnostic Systems Inc., Medford, NY), have been used for the detection of TB in meerkats (Drewe *et al*. 2009b). The BovidTB Stat-Pak Assay was reported to have a low diagnostic sensitivity and a reasonably high specificity.

## Evolution of the African members of the *Mycobacterium tuberculosis* complex

The ancient common ancestor of the MTC is believed to have been a pathogen that originated in East Africa and spread with the global migration of humans (Comas *et al*. 2013; Gutierrez *et al*. 2005). The clonal evolution of this organism has resulted in distinct MTC lineages that are marked by a number of significant genetic deletions and SNPs, some of which may have been pathoadaptive (Namouchi *et al*. 2012). Such changes may have improved the fitness of particular lineages through the modification of traits involved in survival within the host and allowed for the infection of, and establishment in, novel host niches (Namouchi *et al*. 2012). Notably, the lineage characterised by the loss of RD9 is the only clade with distinct strains that are associated with both humans, that is, *M. africanum*, and a wide range of animal hosts (Brosch *et al*. 2002).

Interestingly, the three MTC members reviewed here show a genetic deletion within the RD1 locus, i.e. RD1^das^, which contains genes that encode culture filtrate protein 10 kDa (CFP-10) and early secretory antigenic target 6 kDa (ESAT-6). Both proteins are highly immunogenic T-cell antigens and potent mycobacterial virulence factors (Alexander *et al*. 2010; Mostowy *et al*. 2004; Parsons *et al*. 2013). MTC strains with RD1 deletions have been associated with decreased bacterial proliferation, reduced systemic dissemination and decreased host pathology (Lewis *et al*. 2003). However, such attenuated virulence may be host specific as the dassie bacillus has low virulence in guinea pigs and rabbits but is capable of causing TB in hyraxes (Mostowy *et al*. 2004; Wagner & Bokkenheuser 1961). Therefore, the interplay between the host immune response and the ability of a pathogen to evade this response will ultimately determine whether or not inter-host transmission occurs as a short-lived spillover event or as a self-sustaining infection (Pedersen & Davies 2009). Indeed, cross-species infection is more likely to occur between closely related hosts which exhibit similar immunological responses to particular pathogens (Pedersen & Davies 2009). Such a phenomenon has been shown by the occurrence of *M. mungi* and *M. suricattae* in mongooses and meerkats respectively, both members of the Herpestidae family.

In addition to the genetic background of both host and pathogen, ecological factors may contribute to the establishment of a pathogen in a novel species (Killiny & Almeida 2011). These include the geographic distribution, population density and contact rates of potential hosts (Pedersen & Davies 2009). Moreover, behavioural and life history traits such as a gregarious nature, burrow-living, grooming, scavenging and longevity may influence disease epidemiology. Notably, the host species reviewed here are all gregarious and territorial and have lifespans of approximately 12 years (Skinner & Chimimba 2005). Meerkats and mongooses display social grooming, and meerkats are known to utilise the burrows of other species such as yellow mongooses (*Cynictis pencillata*) and ground squirrels (*Xerus inauris*). Hyraxes may also inhabit abandoned burrows of other animals (Skinner & Chimimba 2005).

## Conclusion

The occurrence of three indigenous southern African members of the MTC that cause endemic TB in hyraxes, mongooses and meerkats is of notable ecological and evolutionary interest. However, while TB is known to have contributed to local extinctions of isolated mongoose and meerkat groups, it is currently unclear what risk these organisms may pose to other populations and species.

Importantly, the emergence of a potentially fatal disease can result in a loss of genetic variation in affected host populations as well as wider ecological effects on predators of the host and on plants or animals on which the host feeds (Daszak, Cunningham & Hyatt 2000). Small mammals such as mongooses, meerkats and hyraxes are prey for a number of African predators, including caracals (*Caracal caracal*), wild dogs (*Lycaon pictus*), jackals (genus *Canis*) and leopards (*Panthera pardus*) (Skinner & Chimimba 2005). Moreover, the host species reviewed here are readily habituated to human activity and this trait carries a zoonotic risk and risk to livestock.

For these reasons, awareness of the southern African members of the MTC is important in order to mitigate anthropogenic disturbances that might introduce these pathogens into novel populations or increase disease incidence in already affected groups. In particular, the translocation of hyraxes, mongooses and meerkats from populations known to be infected with or exposed to members of the MTC should be undertaken with caution. This has been demonstrated by the exportation of MTC-infected hyraxes to Canada (Lutze-Wallace *et al*. 2006), Australia (Cousins *et al*. 1994) and Croatia (Gudan *et al*. 2008).

In order to prevent such events, the detection of infected individuals is required. Direct detection of mycobacteria by bacterial culture and/or polymerase chain reaction (PCR) analysis is the most specific method for confirming infection. However, these techniques are insensitive in individuals with limited pathology (Drewe *et al*. 2009b). Moreover, a number of genotyping methods commonly used for members of the MTC are not suitable for identifying the mycobacterial strains reviewed here (Alexander *et al*. 2010; Parsons *et al*. 2013). Therefore, in cases in which infection with these organisms is suspected, genetic speciation techniques should target the unique genetic deletions or polymorphisms of these strains. PCR analysis for the presence or absence of RD1, RD4, RD9 and RD12 will differentiate *M. bovis* and *M. tuberculosis* from the three South African MTC members (Warren *et al*. 2006). In addition, detection of the N-RD25^das^ deletion will differentiate these strains from all other MTC organisms (Mostowy *et al*. 2004). MIRU-VNTR analysis (Table 1) and spoligotyping (Figure 3) can be used to differentiate between the three southern African strains; however, more specifically, *M. suricattae* can be identified as having a unique *16S rRNA* gene sequence (Parsons *et al*. 2013); *M. mungi* can be confirmed by the absence of the SNP Rv0911^398^ (Alexander *et al*. 2010); and the dassie bacillus confirmed as having a typical RD5^das^ deletion (Mostowy *et al*. 2004; Parsons *et al*. 2013).

A more sensitive test of mycobacterial infection is the detection of a host’s immune response, especially of cell-mediated immunity, to mycobacterial antigens. However, to date, in the species reviewed here, only a single commercial assay of humoral immunity, namely the BovidTB Stat-Pak Assay, has been evaluated in meerkats (Drewe *et al*. 2009b). In this species, this assay appears to have limited sensitivity. Nonetheless, meerkats have been shown to develop immune responses to a number of mycobacterial antigens. These include Acr1/MPB83 fusion protein, 38 kDa protein and MPB59, which might be used as immunodiagnostic antigens (Drewe *et al*. 2009b). Moreover, 19% of culture-positive meerkats were serologically reactive to ESAT-6. This finding is surprising given that the gene encoding this protein has been deleted from the *M. suricattae* genome (Drewe *et al*. 2009a; Parsons *et al*. 2013).

In summary, the risks associated with endemic MTC infections in hyrax, mongoose and meerkat populations in southern Africa are currently unknown. Further studies are essential for understanding this disease in wildlife populations and developing strategies for its potential management. In order to achieve this, sensitive diagnostic tools are needed to detect infected individuals and to prevent the translocation of disease.
